# Sonographic Detection of a Torsed Meckel’s Diverticulum Misinterpreted as Acute Appendicitis

**DOI:** 10.5811/cpcem.2019.5.42976

**Published:** 2019-07-22

**Authors:** Justin Choi, Nicole Dorinzi, Justine Pagenhardt, Anthony Steratore, Melinda Sharon, Joseph Minardi

**Affiliations:** West Virginia University, Department of Emergency Medicine, Morgantown, West Virginia

## Abstract

A 38-year-old female presented to the emergency department (ED) with acute-onset right lower quadrant abdominal pain following two days of nausea and vomiting. Physical examination revealed right lower quadrant tenderness to palpation, rebound tenderness, and guarding. Point-of-care ultrasound (POCUS) of the right lower abdomen was performed and interpreted as probable appendicitis. However, upon laparoscopic examination of the abdomen, a benign-appearing appendix was visualized. Further investigation revealed the source of the patient’s pain to be a torsed Meckel’s diverticulum. Although rare, a torsed and inflamed Meckel’s diverticulum can be visualized by POCUS in the ED without the need for further imaging or delay.

## INTRODUCTION

Acute abdominal pain is a common presentation in the emergency department (ED). Pain localized to the right lower quadrant (RLQ) warrants a workup for appendicitis, a diagnosis commonly made in the ED and a prevalent cause of urgent abdominal surgery.^1^ Imaging of the abdomen in suspected appendicitis is important for distinguishing true appendicitis from appendicitis mimics in order to prevent negative appendectomies and unnecessary surgical risks. However, certain mimics warrant surgical treatment. One example is an inflamed or torsed Meckel’s diverticulum that is symptomatic.^2^ While the imaging modality of choice for RLQ pain suggestive of acute appendicitis has been computed tomography (CT) of the abdomen and pelvis, many ED providers are moving towards abdominal ultrasound (US) as the initial imaging study, as it can identify both appendicitis and, as in this case, a torsed Meckel’s diverticulum at the bedside without the need for radiation exposure.^3^

## CASE REPORT

A 38-year-old white female with no significant past medical history presented to the ED with acute onset RLQ abdominal pain that began earlier that day. The pain was described as being sharp, stabbing, radiating to the right flank, and greatly exacerbated by movement. She reported decreased appetite, nausea, and vomiting two days prior to presentation with only nausea on the day of admission. On physical examination, the RLQ of her abdomen was tender to palpation with guarding and rebound tenderness. Other examination findings included a blood pressure of 90/60 millimeters of mercury, a heart rate of 97 beats per minute, and a temperature of 36.8 degrees Celsius. Pertinent laboratory findings included leukocytosis, with a white blood cell count of 17.6 *10^3^/microliter, a normal hepatic and pancreatic function panel, a normal basic metabolic panel, and a negative pregnancy test.

Based on the patient’s presenting symptoms and workup, a point-of-care ultrasound (POCUS) of the abdomen focused on the RLQ was performed to assess for suspected appendicitis. A linear probe was used on the RLQ to first obtain a short-axis view, and then rotated 90° to obtain a long-axis view. The bedside US interpreted by the emergency physician showed a tubular, non-peristalsing structure superficial to the iliac vessels 0.9 centimeters in diameter with edematous walls consistent with appendicitis (Image, Video). Further increasing the likelihood of appendicitis was the structure’s “target” appearance on short-axis, associated with the gut signature of bowel wall (Image, Video).

Surgery was promptly consulted for acute abdomen with a preoperative diagnosis of appendicitis. The patient was consented for a laparoscopic appendectomy and taken into the operating room. Upon initial laparoscopic examination of the lower abdomen, the patient’s appendix appeared normal, with no signs of appendiceal inflammation, induration, or injection. The appendix was removed despite its normal appearance, and the lower abdomen was again examined for another source of the patient’s pain. An area of torsed, dark tissue was identified on the antimesenteric portion of the small bowel and subsequently detorsed, revealing an ischemic Meckel’s diverticulum that appeared to be the cause of the patient’s symptoms. The transected tissue was sent to pathology, which confirmed both a normal appendix and a Meckel’s diverticulum. The patient recovered and was discharged two days later with weight restrictions and home rest for two weeks.

## DISCUSSION

Meckel’s diverticulum is the most common congenital anomaly of the gastrointestinal tract, with a prevalence of 2–4%.^4^ Meckel’s diverticulitis and torsion of a Meckel’s diverticulum is often clinically, and in many cases, radiologically difficult to distinguish from appendicitis. Meckel’s diverticulitis is accurately diagnosed preoperatively in fewer than 10% of patients, with acute appendicitis being the most common preoperative diagnosis.^5^–^6^

**CPC-EM Capsule**

What do we already know about this clinical entity?

Meckel’s diverticulum is the most common congenital gastrointestinal anomaly. When torsed or inflamed, it can clinically and radiologically mimic acute appendicitis.

What makes this presentation of disease reportable?

A rare, torsed Meckel’s diverticulum was thought to be appendicitis but properly treated after visualization via point-of-care ultrasound.

What is the major learning point?

Visualization of the pathological process is more crucial to patient care than distinguishing an inflamed Meckel’s diverticulum from appendicitis.

How might this improve emergency medicine practice?

Point-of-care sonography can save time and resources as well as reduce radiation exposure without negatively affecting outcomes in right lower quadrant pain.

Many factors contribute to the difficulty in correctly distinguishing between a torsed or inflamed Meckel’s and an inflamed appendix. Both can present with a similar history of acute onset right lower abdominal pain with variable abdominal distention, nausea and vomiting, especially in the case of bowel obstruction with Meckel’s diverticulum.^7^ Laboratory findings are also relatively nonspecific, with both appendicitis and any diverticulitis commonly presenting with leukocytosis and fever.^8^ Physical examination findings commonly show RLQ tenderness on palpation as well as guarding, rigidity, and rebound tenderness. While pain from Meckel’s diverticulum can be located more toward the midline, periumbilical pain is common due to peritoneal irritation with early acute appendicitis as well, rendering this finding nonspecific.

Regarding imaging findings, torsion of a Meckel’s diverticulum is an extremely rare complication and thus there are limited studies of optimal imaging modalities.^6^ In the ED, the most likely scenario is high suspicion of acute appendicitis, with a torsed Meckel’s diverticulum being near the bottom of the differential diagnosis if even considered. Thus, it is fitting to deliberate over imaging options in the context of acute appendicitis. Historically, the initial imaging modality of choice for suspected appendicitis has been CT, with alternative modalities such as magnetic resonance imaging or US reserved for children and pregnant women due to concern for radiation exposure.^9^ However, many emergency physicians are moving toward abdominal US as the initial imaging modality for suspected acute appendicitis, typically via the high-frequency linear probe, with sensitivity and specificity reaching values above 90%.^3^,^10^

US can also visualize a torsed Meckel’s diverticulum, as shown in this case; however, it is often misdiagnosed as acute appendicitis due to similar location, features, and anatomy. Anatomically, Meckel’s diverticulum is classically found within 100 centimeters of the ileocecal valve, placing it in the RLQ or near the midline, analogous to the appendix. US findings are also similar between the two diagnoses. Both are true diverticula meaning they contain all gut wall layers, resulting in the classic gut signature or “target” appearance on ultrasonography, and both show non-compressible, blind-ending, tubular, non-peristaltic structures with edematous walls (Image).

While it is possible to correctly identify a Meckel’s diverticulum on ultrasonography by its lack of association with the cecum, this case shows that from an ED perspective, the visualization of a pathological process that is likely the source of the patient’s pain is much more crucial and meaningful than accurately diagnosing a Meckel’s diverticulum at the bedside. The aforementioned sonographic findings coupled with the patient’s presenting symptoms would warrant surgical consultation in either case. Whether the findings are indicative of appendicitis or a torsed Meckel’s diverticulum can be demonstrated intraoperatively, as both of these etiologies would require surgery.

Here, we see that US is a valuable study for RLQ pain even in the setting of bowel ischemia secondary to axial torsion of a Meckel’s diverticulum. In this particular case, a torsed Meckel’s diverticulum was mistakenly identified as appendicitis, but the patient’s treatment course and outcome were not affected. On the contrary, the patient was quickly consented for laparoscopic surgery without the need for further imaging, interpretation by a radiologist, or exposure to unnecessary radiation.

## CONCLUSION

Few studies have investigated the utility of ultrasonography in the setting of an axially torsed Meckel’s diverticulum. Here, we have shown that POCUS in the ED is a capable and appropriate tool for investigating acute RLQ abdominal pain even in the setting of a Meckel’s diverticulum. Although US is relatively operator-dependent, in the right hands it is a readily available and quickly interpreted imaging modality, making it an important skill for emergency physicians.

## Supplementary Information

Video. A linear probe is being used on the right lower quadrant of the abdomen in this video clip. An edematous, fluid-filled, non-compressible structure is seen just below the abdominal wall musculature in short-axis view and is circled in yellow. The structure displays a “targetoid” gut signature and is shown adjacent to normal loops of small bowel, making it suspicious for acute appendicitis. It was later found to be an inflamed Meckel’s diverticulum.

## Figures and Tables

**Image:**
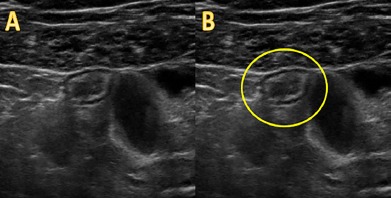
*Frame A* is a short-axis view of a point-of-care ultrasound showing an edematous, non-compressible tubular structure in the right lower quadrant of the abdomen with adjacent normal-appearing, compressible loops of small bowel. Clearly visible is the “gut signature” associated with the different layers of the bowel wall; serosa (echogenic), muscularis externa (hypoechoic), submucosa (echogenic), muscularis mucosa (hypoechoic), and mucosa (echogenic). This structure is circled in *Frame B*.
